# Sorting rare earth magnets motors for recycling without opening the motors

**DOI:** 10.1038/s41598-025-94667-x

**Published:** 2025-04-10

**Authors:** A. P. S. Baghel, A. Karati, D. Prodius, I. C. Nlebedim

**Affiliations:** Critical Materials Innovation Hub, Ames National Laboratory, US DOE, Ames, IA 50011 USA

**Keywords:** Rare earth elements (REEs), Electric motor recycling, Permanent magnet motors, Power density sorting, Sorting motors without opening, Energy science and technology, Renewable energy, Materials for energy and catalysis, Techniques and instrumentation

## Abstract

A novel approach for sorting electric motors without dismantling them is reported in this article. This approach increases the likelihood that a motor selected from a mixture for recycling would contain rare earth elements (REEs), which are pivotal for numerous advanced green technologies. The challenge of inadvertently dismantling motors without REEs during recycling is addressed by this proposed innovative method. It relies on cogging interactions and power density for sorting motors with REEs. The process uses an algorithm introduced in this work to sort motors in two steps by (a) distinguishing induction motors from permanent magnet motors, and (b) distinguishing motors with critical REEs (e.g., Nd, Pr, Dy, Tb) from ferrite-based motors, all without opening the motors. The former was accomplished with 100% accuracy while the latter was accomplished with ~ 78% accuracy. Such high accuracy improves the recycling efficiency of critical REEs from motors, alleviating concern about supply disruption and supporting resource conservation and sustainability.

## Introduction

**Fig. 1:**
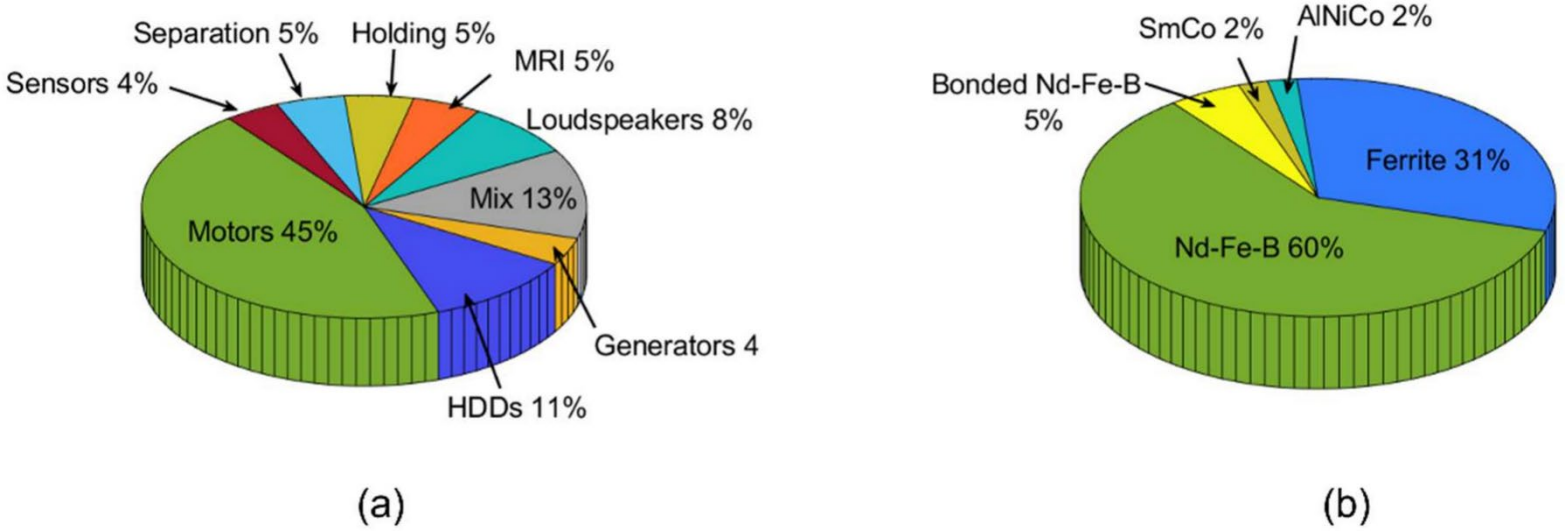
(**a**) Percentage of market share for different permanent magnet applications^1,9^ (**b**) Percentage of market share by value for different permanent magnet types^9^.

Rare earth permanent magnets (REPMs) are becoming the key to emerging green technologies, including those that enable transportation electrification and renewable energy infrastructure, which are currently attracting global attention^1–3^. The best magnetic properties among all commercially available permanent magnets (PMs), in terms of energy density, are achieved with Nd-Fe-B magnets^4^, making them beneficial for a wide range of applications, particularly in clean transportation and other green energy technologies^5,6^. Moreover, Nd-Fe-B magnets are also used in high-performance industrial motors, hard disk drives (HDDs), headphones, speakers, washing machines, and most consumer electronic devices^7^. Figure 1a shows the distribution of the use of Nd-Fe-B magnet among various applications. The magnets contain around 30 wt.% of REEs which, in turn, accounts for ~ 70% of the total cost^8^. Figure 1b shows that the permanent magnet market is dominated by Nd-Fe-B magnets, occupying 60% of the market share by value. Of the REEs, neodymium (Nd), praseodymium (Pr), dysprosium (Dy), and terbium (Tb), are responsible for the excellent magnetic properties of Nd-Fe-B magnets^2^. Dysprosium and terbium are the key elements for improving magnet performance at higher temperatures in electromagnetic devices^7^.

REE supply and demand have been a major concern in recent years and attracted global attention due to highly concentrated supply sources, concern for disruptions in supply chains, and environmental impacts of mining^10,11^. This is particularly relevant to the REEs used for permanent magnets production. Therefore, REE recycling from end-of-life (EOL) products is becoming an inevitable option to ease the demand for critical REEs^12,13^. Magnets from EOL devices can be efficiently recycled as a means of resource conservation for REEs^14,15^. However, REEs recycling from magnets is still in its early stage, and some of its major challenges are insufficient recycling feedstock due to limited collection of EOL products, wide variations in REE contents in different applications, different lifespans, and lack of standardized recycling processes for different EOL products^3,14^. There are also challenges associated with establishing a sustainable urban infrastructure system to balance the interplay between raw materials production, resource consumption, waste generation and recycling. Such a system would help to predict the availability of REE from EOL products.

One way to address the above-mentioned challenges has been proposed, the so-called infrastructure ecology^16^. Another way to address the challenges is to establish the ability to predict feedstock materials’ availability and concentration from different application groups^17–19^. Gaustad et al. has reported on the availability of REEs from various secondary sources, including electronic waste^20^. Electric motors can be significant secondary sources of REEs for recycling because they are widely used in applications, particularly in electric mobilities, personal/home devices and industrial automation. They account for 45% of all the Nd-Fe-B applications (Fig. 1a). However, recent literature reports state that less than 3% of high-value REEs used in electric motors are recycled globally^21,22^. Hence, there is need to invest in creating value recovery plans from electric motors^23^ and procedures for mitigating the criticality and potential disruptions in the supply of important REE materials^24^. This will play a significant part in building a supply chain that is well-positioned for the future. Moreover, the diverse types of electric motors (some of which contain REPM, and some do not) necessitate the need for a pre-processing step, such as sorting and dismantling^25^. Furthermore, electric motors used in high-power applications, such as e-mobility and wind energy, are typically known to include detailed information about the permanent magnets and the cooling arrangements used in their designs^26^. However, motors in low-power applications often lack such detailed information, making it more challenging to identify the type and presence of magnets in these motors. Presently, no commercial operations have been identified that involve pre-processing, such as sorting and dismantling EOL motors, to improve value recovery through recycling. Such preprocessing operation would require prior knowledge of magnet types in motors, otherwise recycling efficiency would be limited by the inadvertent dismantling of the motors that do not contain REEs.

Fig. S1 shows the RE recycling scheme which can be divided into four stages. The first stage is the collection and transportation to the recycling plant location which requires information about application groups and concentrated locations of EOL products^19^. The second stage is the pre-processing of EOL products which includes screening, dismantling, and cleaning of magnets depending on the recycling strategy to be applied^27–31^. In the recycling stage (third stage) scrap magnets can be recycled by choosing an appropriate strategy from direct reuse, reprocessing, or elemental recovery^14,32–34^. Depending on the strategy chosen, the recycled products can be in the form of powders, dense magnets, or rare-earth oxides (the fourth stage). Thus, successful recycling relies both on the strategy chosen (reuse/reprocessing/elemental recovery)^3,14,35^, and the adopted pre-processing approach (e.g., screening, dismantling, demagnetization, etc.) to obtain the magnet feedstock from EOL devices^27–29^. In addition, it is critical for the adopted approaches to be based on frameworks that maximize value recovery in a manner that is both economically feasible and environmentally sustainable.

The main motivation of the present study is to introduce pre-processing steps for sorting and classifying of EOL devices, specifically motors, to enhance rare earth value recovery. In addition, high-quality copper, electrical steels, aluminum, etc. in e-mobility applications can be recovered in metallic forms which have higher values than oxides or salts^21^. On the other hand, the use of manual dismantling as part of the pre-processing steps may be cost-prohibitive in places with high labor wages such as the USA and Europe. This calls for automation, which, in turn, is difficult due to different rotor designs (surface and interior mounted magnets), strong magnetic forces, and brittleness of magnets. Also, due to new lightweight and compact motor designs, disassembly and dismantling might be a more difficult and time-consuming process. During pre-processing of EOL motors, the likelihood of inadvertent dismantling of non-REPM motors, alongside REPM motors can add additional expense. This likelihood arises because the development of motors can be satisfied with REPM, ferrite and AlNiCo magnets, or even without any magnet, such as in induction motors^36–38^. For example, 23% of e-mobility applications are not based on REPMs (Fig. S2)^36^. Therefore, EOL PM motors may also contain magnets that are not based on critical rare earths, such as ferrite magnets, particularly for small industrial motors, and two-wheeler motors^37–39^. Consequently, recycling efficiency can be improved if pre-processing screening approaches can be established to increase the likelihood that the dismantled EOL motors are based on critical rare earths. Implementing a screening procedure of this kind can also reduce contamination in subsequent recycling stages. However, such a screening process needs to be developed for efficient REEs recycling practices.

This work aims at offering a novel preprocessing technique that identifies the magnet-types in electric motors without dismantling them. It involves evaluating the physical condition of EOL motors (e.g., winding resistance, insulation, etc.) for electrical testing or rating information needed to calculate their power density. Next, the presence of permanent magnets is checked by rotating the rotor shaft at a controlled speed (without energizing the motor) to detect the cogging interaction between the permanent magnet and stator. Following this initial test, the next step is to determine whether the motor contains REE magnets, using a screening algorithm that relies on the power density (PD) value to differentiate between REE and non-REE magnet EOL motors. Therefore, this work minimizes inadvertent dismantling of non-REPM motors, hence decreases the contaminations in the REE manet recycling feedstock. This technique offers improved sorting of various EOL motors for more efficient and faster REE recovery.

## Results

### Validation of the screen process

The method established in this work was validated on 16 EOL motors of which it was unknown whether they are induction motors or motors based on permanent magnets. First, by slowly and manually rotating the shaft of the 16 scrap motors, 7 of them were determined to be non-permanent magnet motors using the cogging interaction between the magnets and the stator of the motors. The remaining 9 motors were determined to be based on permanent magnets and were further screened using their PD values to distinguish motors that contain rare earth magnets from those that contain non-rare earth magnets (i.e. ferrites).

**Fig. 2:**
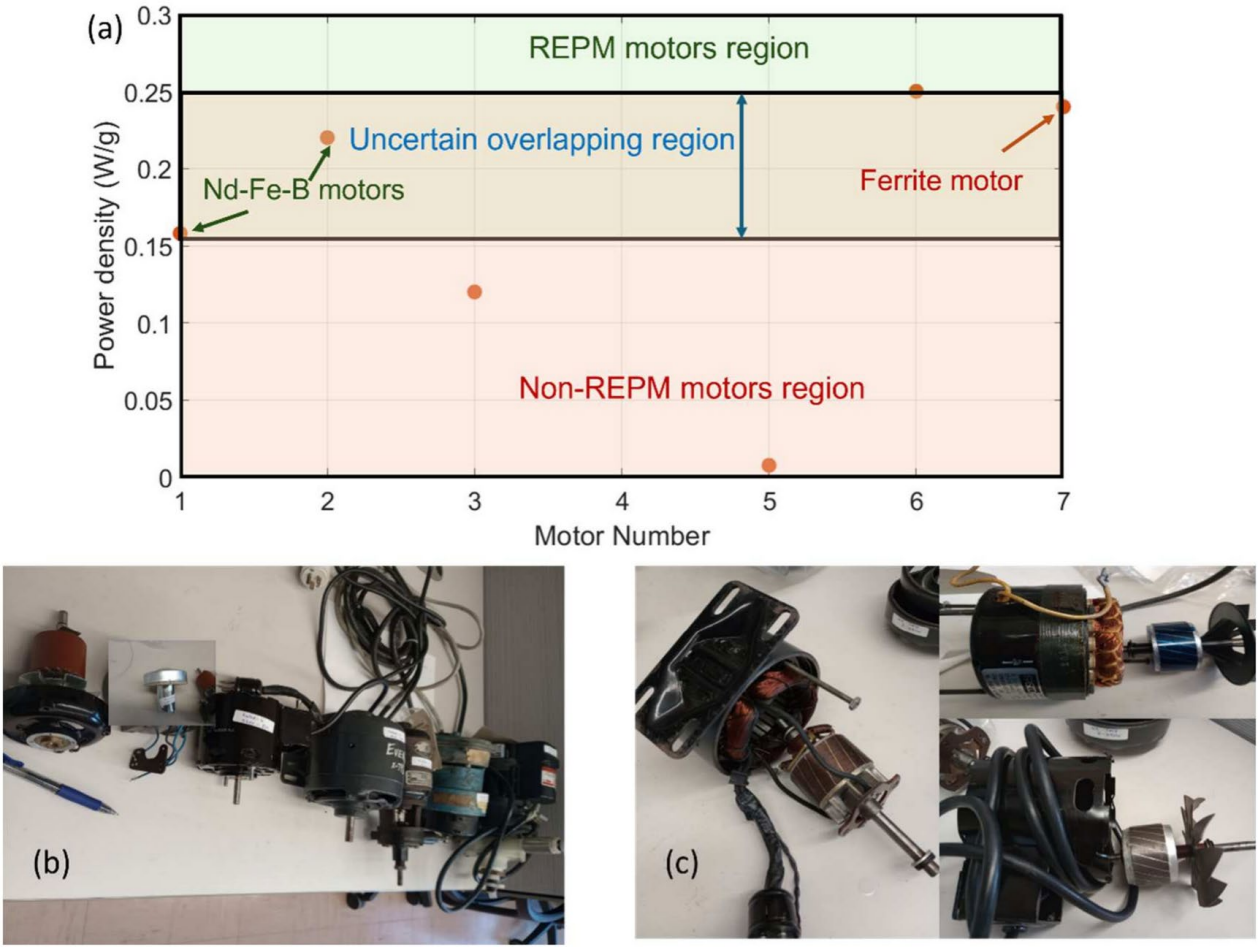
(**a**) PD values boundaries for screening REPM from non-REPM motors, including an uncertain region. EOL motors that were used to validate the PD value boundaries established in the work: (**b**) Prior to dismantling, and (**c**) Dismantled induction (i.e. non-PM) motors.

The result of classifying the motors based on their PD values was guided by magnetic thermogravimetric analysis (mTGA) and X-ray fluorescence (XRF) spectroscopy. Using the mTGA and XRF results, the PD values boundaries were established as shown in Fig. 2a. 100% REPM region would correspond to $PD\ge 0.25 W/g$ while the 100% non-REPM/ferrite region corresponds to $PD\le 0.15 W/g$. The uncertainty region lies between these two values. It is worth noting here that testing more motors can improve the accuracy and reduce the uncertainty region.

Afterwards, the motors were dismantled to validate the methodology. Figure 2b shows the motors prior to dismantling, while Fig. 2c shows the dismantled motors. Since cogging interaction can only be detected in non-energized motors if they are based on permanent magnets, the induction motors were sorted from permanent magnet motors with 100% accuracy. The accuracy of sorting the REPM from the non-REPM motors using the screening algorithm was 77.8%, i.e., 7 of the 9 motors were correctly predicted. The accuracy level can be increased by minimizing the uncertainty region. One potential approach is to apply this method to a larger number of motors.

## Conclusions

The approach used in the present work increases the likelihood that a PM motor being recycled would contain REEs. It enhances the viability of recycling EOL products as a means for addressing supply chain resilience for REEs and fostering the sustainability of the dependent clean energy applications. The approach begins by sorting out non-PM motors, such as induction motors, and then uses a screening algorithm to differentiate PM motors based on magnet type (RE vs. non-RE) using PD. The former was accomplished with 100% accuracy while the latter was accomplished with ~ 78% accuracy, significantly improving recycling efficiency. A limitation of the current approach, to be addressed in future studies, is the need to refine the method by establishing clear power density (PD) boundaries for EOL motors from different applications, which would enhance the sorting of REPM motors across various applications. Additionally, to minimize uncertainty, it could be beneficial to leverage the collected data to train machine learning models, facilitating a more accurate sorting method across a wider range of applications. Furthermore, it is worth noting that the process outlined in this work has the potential for automation, which could help enhance throughput significantly during scale-up. Additionally, the rotation of the motor shaft can be automated to further streamline the process, without the need to energize the motor. This approach could be further extended for automated dismantling by integrating a recently reported technique^40^ to also identify the motor’s topology.

## Methods

**Fig. 3:**
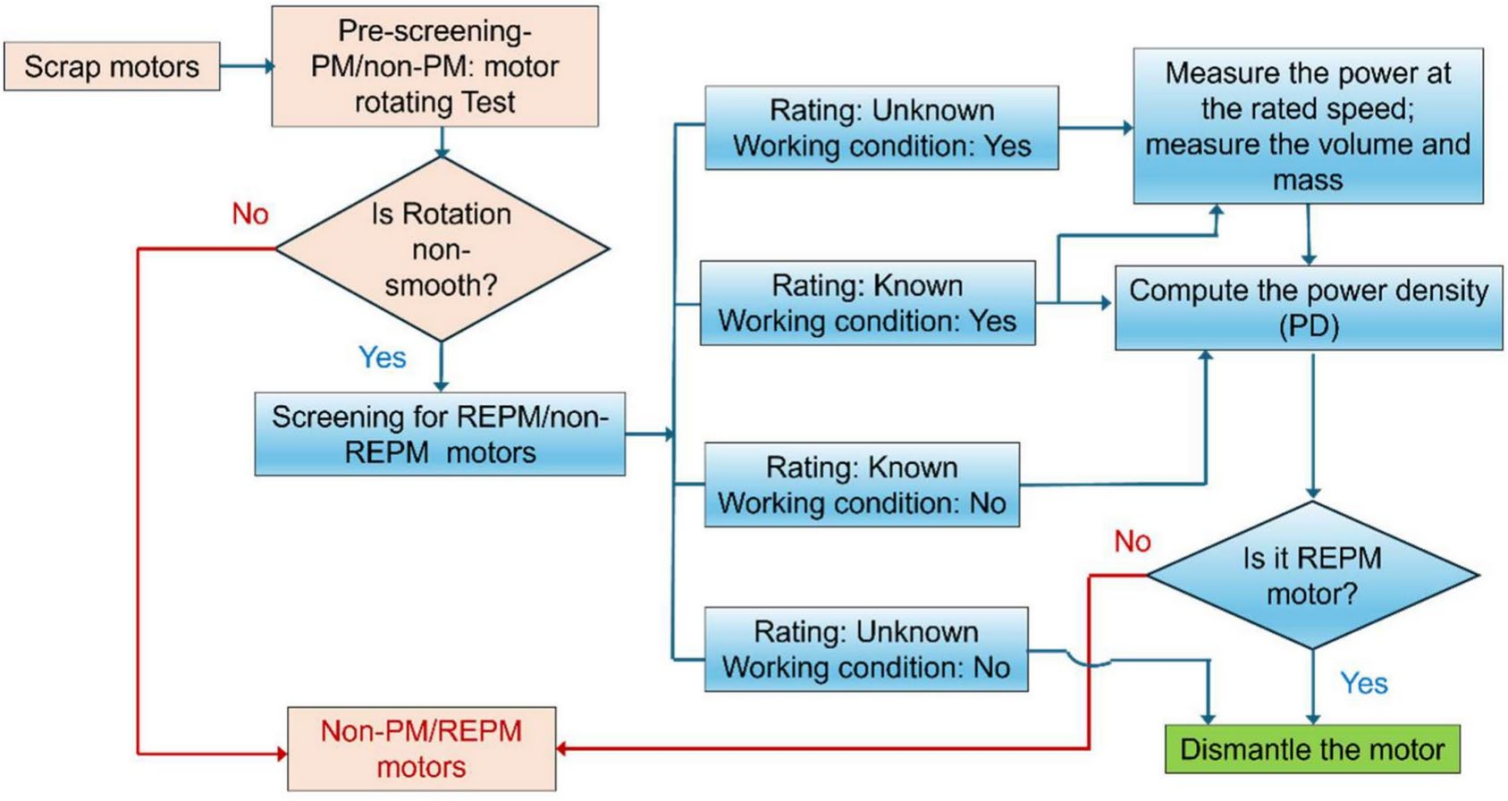
A screening algorithm developed for sorting REPM motors from a mixture of motors, aimed at increasing the likelihood that a dismantled motor would contain REEs.

The flow process for the sorting algorithm reported in this work is shown in Fig. 3. The first step is to determine whether the motor contains PM, irrespective of which type (REPM or non-REPM). The objective is to separate induction motors and avoid dismantling them for REPM recycling since they do not contain magnets. This is easily done by slowly and manually (i.e., without energizing the motor) rotating the shaft of the rotor such that the cogging interaction between the PM and the stator can be detected. Slow rotation is necessary to ensure that the torque applied to rotate the shaft is less than the resistance to rotation due to the cogging interaction.

Also, manual rotation has been used in this work since such cogging interaction can be present in induction machines, only if the stator windings are energized. Since the permanent magnets in the rotor of REPM machines are magnetized, the cogging resistance can be detected even when the stator coils are not excited. If the rotors rotate smoothly, without the cogging resistance, they can be identified as induction motors. Otherwise, they likely contain PMs (including REPMs and non-REPMs). However, the manual rotation can be mechanized and automated.

**Fig. 4:**
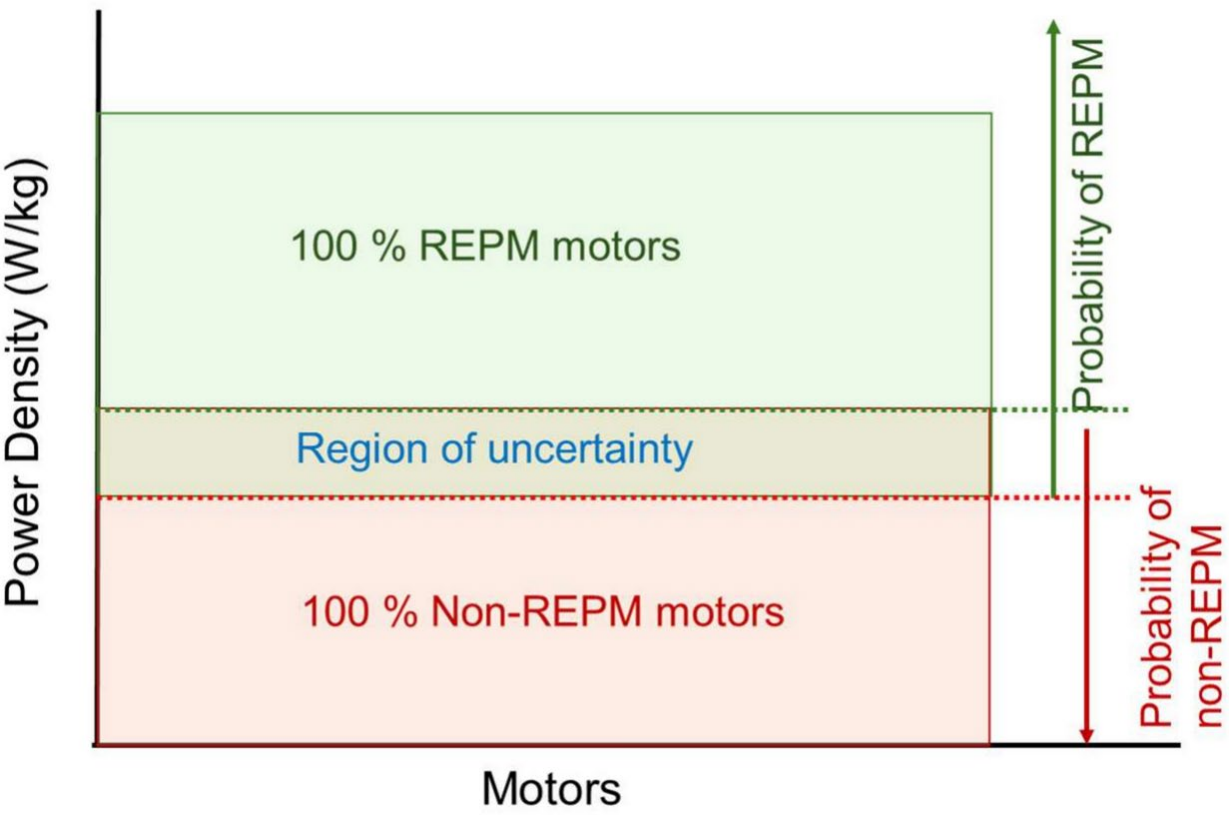
Identification of REPM/non-REPM motors using PD values. REPM motors have higher PD values than non-REPM motors. In the uncertainty region the PDs values of motors with/without REPMs overlap.

After the initial test, the next step is to assess whether the motors contain REPMs. The algorithm uses the power density (PD) value to distinguish REPM from non-REPM devices. Through an appropriate arrangement of rotor permanent magnets, stator slots, and windings, REPMs are widely used in PM motors to achieve higher PD values, compared to induction/non-REPM motors^41–43^. This helps to satisfy the increasing demand for lightweight and compact electric motors^44^ in which torque needs to be maximized, and motor mass minimized. Therefore, the higher the PD value, the more likely that a motor contains REPM^45^, as schematically illustrated in Fig. 4.

PD values can be computed either using the motor power rating (if available) or using the measured values with standard tests (if the motor is in working condition). PD values can vary depending on whether continuous power or rated maximum power is used. Continuous power reflects a motor’s ability to deliver a steady output without overheating, while rated maximum power corresponds to peak performance during short bursts or peak loads. In this study, the rated maximum power was used to calculate PD values, providing an accurate representation of the permanent magnets’ strength. It is important to note that sorting low-power motors is more challenging than high-power motors, as the latter typically have more readily available detailed information^26^. Therefore, this work primarily focuses on low-power motors which generally are air-cooled motors.

Afterwards, motors identified as containing PMs will be subjected to dismantling. One would expect an overlap region in which comparable PD values may create uncertainty in distinguishing motors with REPMs, from those without. In this uncertainty region, the motor could be opened for verification.

### Determination of the PD values for the sorting algorithm

**Fig. 5:**
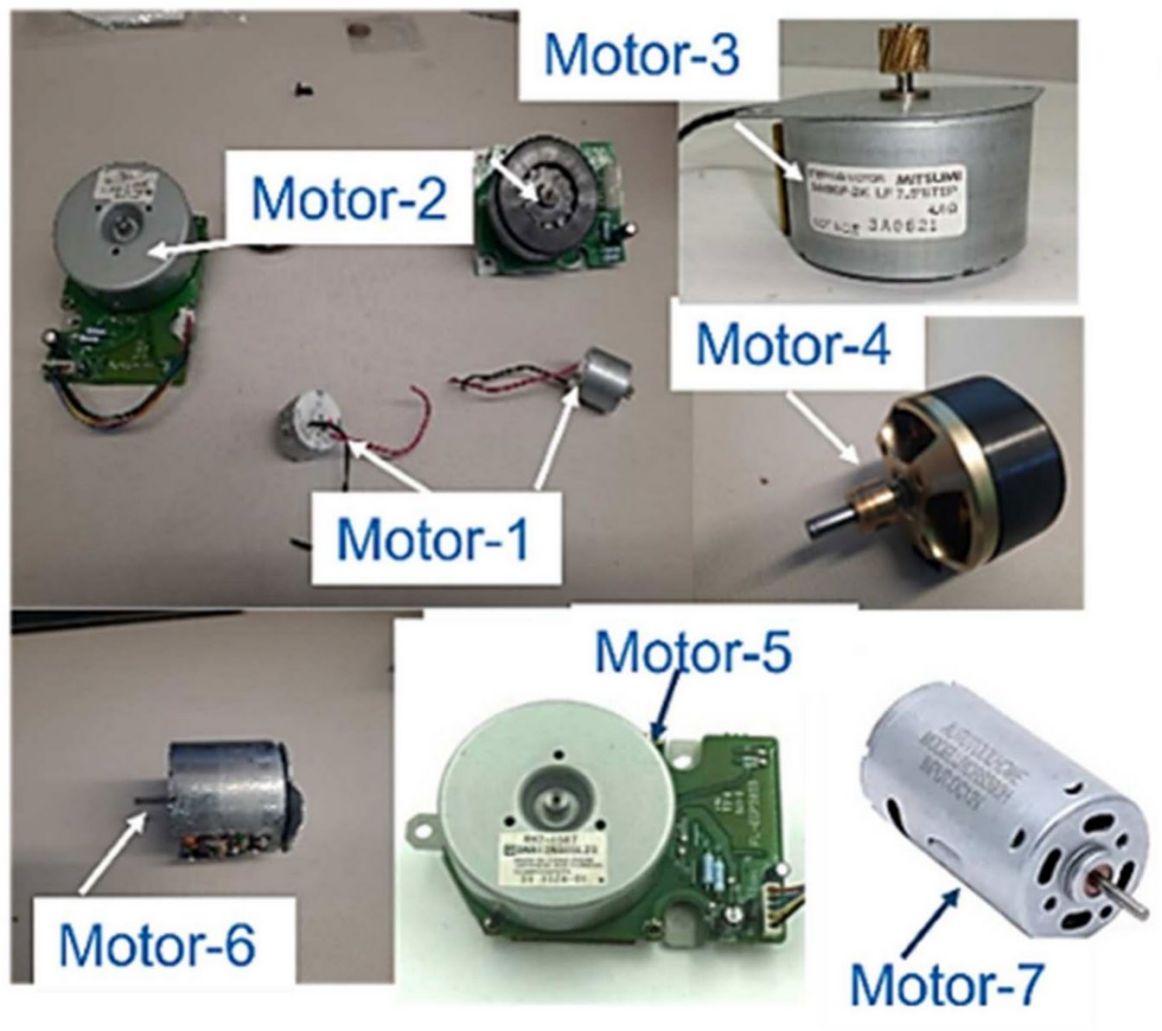
EOL motors that were used to establish the PD values. The description of motors, their PD values, and the types of permanent magnets in the motors are in Table 1.

The non-destructive technique reported here is based on the rotation of the motor shaft and PD, to sort REPM motors from a mixture of EOL motors. To establish the boundaries of the PD values for the screening process, 7 small motors (Fig. 5) were used to develop the screening algorithm. The description of the motors based on their applications, and the determined PD values based on the ratings of the motors are shown in Table 1.

**Table 1:** Scrap motors, magnet types, and their PD values.

Motor No	Applications	PD values (W/g)	PM types based on PD Values
1	Printer, automotive applications	0.15	Nd-Fe-B
2	FAX Printers, multifunction machines	0.22	Nd-Fe-B
3	FAX Printers, copy machines	0.12	Ferrite
4	Small sport airplanes, Toys	4.2	Nd-Fe-B
5	Printer, copy machine	0.076	Bonded-Ferrite
6	Printer, copy machine	0.25	Nd-Fe-B
7	Electric drill, screwdriver	0.24	Ferrite

Using the PD values, the types of PM were assigned to the motors, as also shown in Table 1. As evident, motor #4 has the highest PD value among all the 7 motors, and it is an Nd-Fe-B magnet motor. However, the lower PD value region (applicable to all the motors, except Motor #4) was further analyzed due to the overlapping region between REPM and non-REPM motors. For such analysis, the permanent magnets were harvested from dismantled motors and tested by magnetic thermogravimetric analysis (mTGA) and X-ray fluorescence (XRF) spectroscopy (Bruker M4 TORNADO Micro-XRF spectrometer operated at 50 kV and 300 μA with a Rh target).

The mTGA result obtained for the PMs in the motors were used to classify them based on their Curie temperatures. A pair of permanent magnets attached to the mTGA system creates a magnetic field gradient on the PM samples being analyzed, such that temperature-dependent magnetic order transitions, e.g., Curie temperature (ferromagnetic-to-paramagnetic transition), can be detected as apparent changes in mass^46^. It is quite evident from mTGA results in Fig. S3 that the Curie temperature for magnets in motors (1, 2, 4, and 6) are within the values typical for Nd-Fe-B magnets (300–330 °C)^47^ while the magnets in motors (3, 5 and 7) are near the values expected for ferrite magnets, i.e., 450 °C^47^.

The results of the XRF spectrometry were used to validate that obtained from the mTGA analysis, based on the elemental content of each magnet. For this, the presence/absence of critical rare earth elements (Nd, Pr, and Dy) in the chemical composition obtained from the magnets of EOL motors was used to verify the magnet type. Comparing Fig. S3 and Table 2, the results of both analyses agree.

**Table 2:** XRF results of magnets studied in the present work.

Motor No	Magnet type	Elements (wt.%)				
		Nd	Pr	Dy*	Fe*	Sr
1	Nd-Fe-B	23.80	0.09	0.17	75.94	X
2	Nd-Fe-B	12.25	5.60	ND	82.15	X
3	Ferrite	X	X	X	73.95	26.05
4	Nd-Fe-B	14.93	3.24	2.22	79.61	X
5	Ferrite	X	X	X	69.79	30.21
6	Nd-Fe-B	9.07	7.35	ND	83.58	X
7	Ferrite	X	X	X	72.96	27.04

*ND* means not detected, although the element could be present due to the overlap of the emission lines between Dy and Fe. *X* indicates the element is not present in the composition.

*Overlap of the emission lines between iron and dysprosium results in statistical errors in quantifying them by XRF in a composition in which both are present^48^. Nevertheless, XRF is a simple tool that has been used in the present work to confirm the magnet type, rather than the specific composition.

## Supplementary Information

Supplementary Information.

## Data Availability

Data sets generated during the current study are available from the corresponding author on reasonable request. Determination of a reasonable request and subsequent data provision are subject to institutional approval.

## Abbreviations

REPMs: Rare earth permanent magnets
REEs: Rare earth elements
EOL: End-of-life
PD: Power density
PMs: Permanent magnets
mTGA: Magnetic thermogravimetric analysis
XRF: X-ray fluorescence
HDDs: Hard disk drives
