# Supplementary material for: Sorting rare earth magnets motors for recycling without opening the motors
Source: Sci Rep. 2025 Apr 10;15:12260. doi: 10.1038/s41598-025-94667-x (PMC11985978; doi:10.1038/s41598-025-94667-x)
Supplement: Supplementary file 1 — Supplementary Information. [file 41598_2025_94667_MOESM1_ESM.docx]

**Supplementary Information**

**Sorting Rare Earth Magnets Motors for Recycling Without Opening the Motors**

A. P. S. Baghel, A. Karati, D. Prodius, and I.C. Nlebedim*

Critical Materials Innovation Hub, Ames National Laboratory, US DOE, Ames, IA, 50011, USA

*Author to whom correspondence should be addressed ([nlebedim@ameslab.gov](mailto:nlebedim@ameslab.gov))


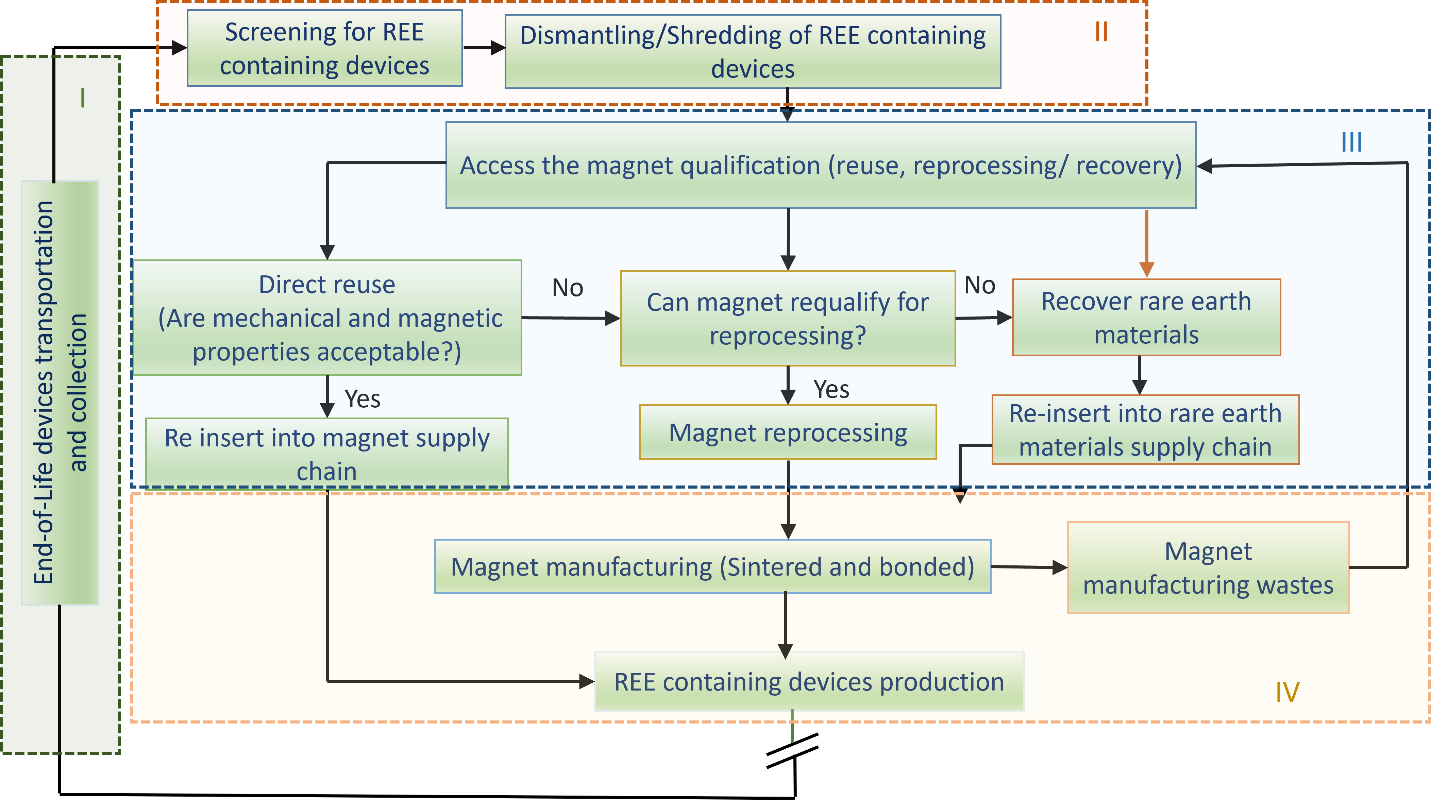


**Fig. S1.** Recycling scheme for RE magnets.


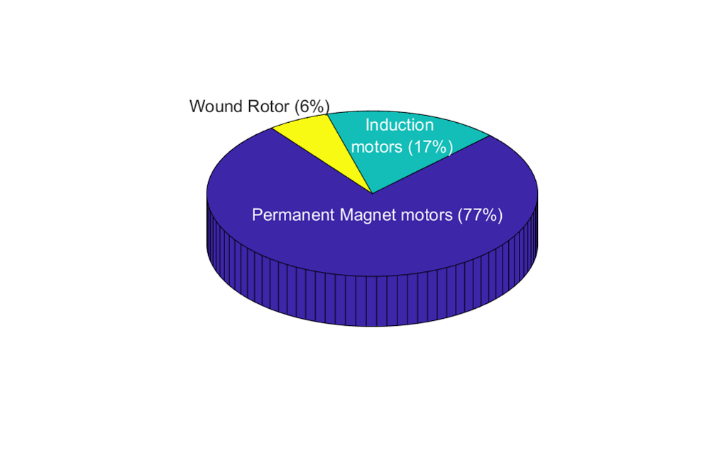


**Fig. S2.** Distribution of PM and non-PM motors in traction applications (Edmondson, 2021).


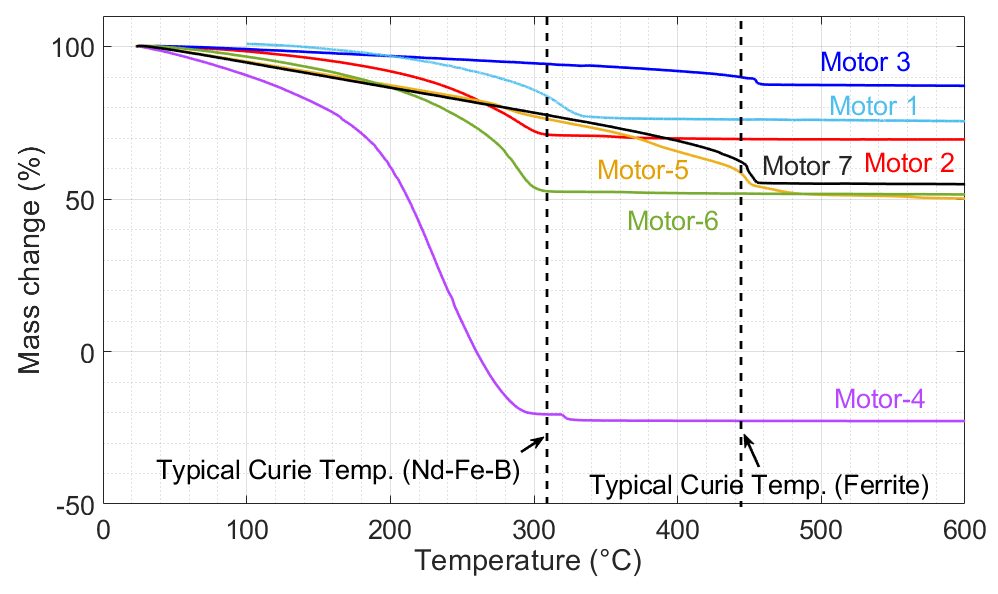


**Fig. S3.** mTGA analysis of scrap magnets from all motors.
